# Supplementary figures and images for: The Assessment of Muscle Mass and Function in Patients with Long-Standing Rheumatoid Arthritis
Source: J Clin Med. 2021 Aug 4;10(16):3458. doi: 10.3390/jcm10163458 (PMC8397223; doi:10.3390/jcm10163458)

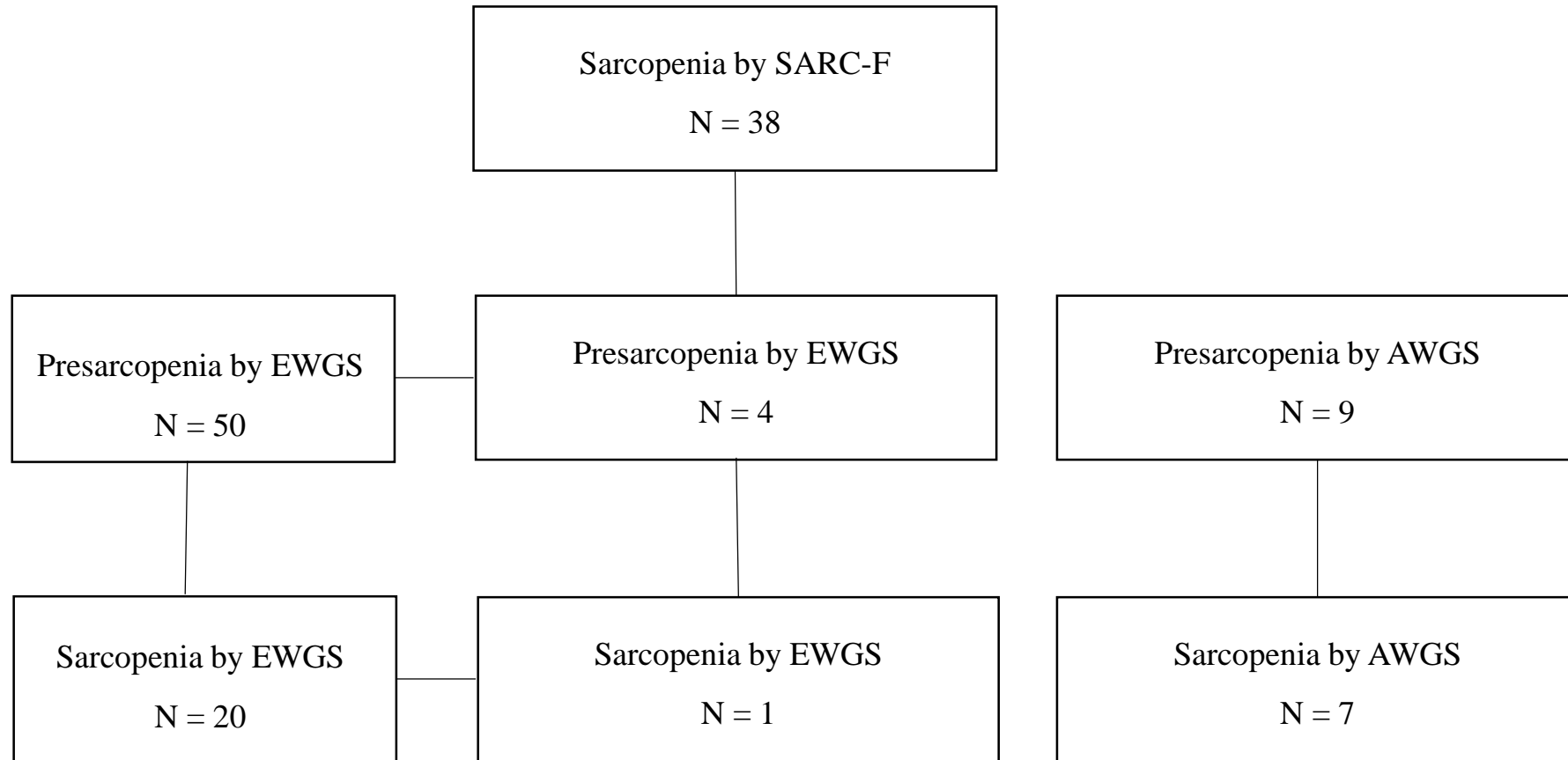

**Figure S1.** Flow diagram.

Supplement: Supplementary file 1 [file jcm-10-03458-s001.zip › jcm-1316585-supplementary.pdf]
